# Dataset on transcriptome signature of skeletal muscle of young, adult and aged mice

**DOI:** 10.1016/j.dib.2022.108321

**Published:** 2022-05-28

**Authors:** Anne Listrat, Kheng Lim Goh, Céline Jousse, Jérémy Tounayre, Huijuan Wang, Kijoon Lee, Daniel Béchet

**Affiliations:** a Université de Clermont Auvergne, INRAE, VetAgro Sup, UMR Herbivores, F-63122 Saint-Genès-Champanelle, France; b Université de Clermont Auvergne, INRAE, UNH, F-63122 Saint-Genès-Champanelle, France; c Newcastle University in Singapore, 172 A Ang Mo Kio Avenue 8 #05-01, 567739 Singapore; d Newcastle University, Faculty of Science, Agriculture and Engineering, Newcastle upon Tyne, NE1 7RU, UK; e Dept of Information and Communication Engineering, DGIST, 333 Techno Jungang-daero, Hyeonpung-Eup, Dalseong-Gun, Daegu 42988, KOREA

**Keywords:** transcriptomic, exon array, muscle, mouse, young, mature adult, ageing

## Abstract

This data article reports the level of expression of messenger RNA (mRNA) obtained from a set of 18 skeletal muscle samples using Affymetrix Genechips Exon arrays. Data were obtained from *Gastrocnemius* muscle of C57BL6 male mice at 3 distinct age groups, 2, 11 and 25 months old representing young, mature adult and aged groups. The data submitted to GEO constitute a large dataset of 15,300 mRNA levels. The data include eighteen .CEL files obtained after scanning mouse exon arrays and one .xls file obtained after processing with Genespring GX 14.9. Three distinct files containing affymetrix data processed using Genespring and analyzed for differences between stages 2 per 2 are provided as supplementary data.

## Specification Table

**Table:** 

Subject	Omics: Genomics and Ageing
Specific subject area	Transcriptomic of muscle growth and ageing
Type of data	Figures, Graphs and Tables
How data were acquired	Affymetrix data obtained from whole-transcript microarray technology (GeneChip Mouse Exon 1.0 ST arrays, Affymetrix Inc, Santa Clara, CA, USA)
Data format	• Raw • Analyzed
Description of data collection	Samples were derived from *Gastrocnemius* muscle of C57BL/6 male mice with three distinct age groups: 2 (young), 11 (adult/mature) and 25 (aged) months (n=6 mice per group) housed in controlled conditions. Mice were anesthetized and samples were taken within 10 minutes after death and flash frozen in liquid nitrogen for subsequent extraction of total RNAs. Total RNAs were isolated using the RNeasy fibrous tissue mini kit (Qiagen). The integrity of the RNAs was verified using an Agilent Bioanalyser with the RNA 6000 Nano labchip® kit. The concentration and purity of the RNAs was determined with a Nanodrop ND-1000 spectrophotometer. The RNAs were hybridized to Mouse Exon 1.0 ST Arrays (Affymetrix). Chips were scanned using a GeneChip® Scanner 3000 7G (Affymetrix).
Data source location	Institution: Institut national de recherche pour l'agriculture, l'alimentation et l'environnement (INRAE) City: Theix Town: Saint Genès Champanelle Country: France Latitude / longitude: 45°42′43.60″ N / 3°0′59.72″ E
Data accessibility	Repository name: NCBI GEO (Gene Expression Omnibus) Data identification number: GSE136266 Direct URL to data: https://www.ncbi.nlm.nih.gov/geo/query/acc.cgi?acc=GSE136266

## Value of the Data

• The main objective of the study was to identify genes or biological processes that were specific to ageing (compared to young age or adulthood). For this reason, this dataset was created to support the need for samples representative of these stages.
• The data were obtained from a set of 18 skeletal muscle samples using Affymetrix Genechips Exon arrays from *Gastrocnemius* muscle of C57BL/6 male mice at 3 distinct age groups, 2, 11 and 25 months old representing young, mature adult and aged groups.
• These data are useful because they can be used to detect specific alterations in mRNA levels that may play a role in growth and ageing mechanisms. These arrays cover the whole genome enabling the potential discovery of previously unidentified events.
• These data are also useful because the arrays used are whole-transcript exon arrays that include multiple probes (about 4) per exon. They enable “exon-level” analysis and investigation of alternative splicing, alternative promoter usage and alternative termination.
• All data are available in two formats: in GEO as Affymetrix .CEL files and as GeneSpring processed .xls file. They can be further processed by researchers using their own bioinformatics algorithms and analyzed together with their own data.
• With the current drive to accelerate transition to a research system that does not involve testing on animals (see go.nature.com/3hzprhj), this dataset will be of primary value to future researchers for reuse without having to repeat the animal testing.

## 1 Data Description

**Fig. 1:**
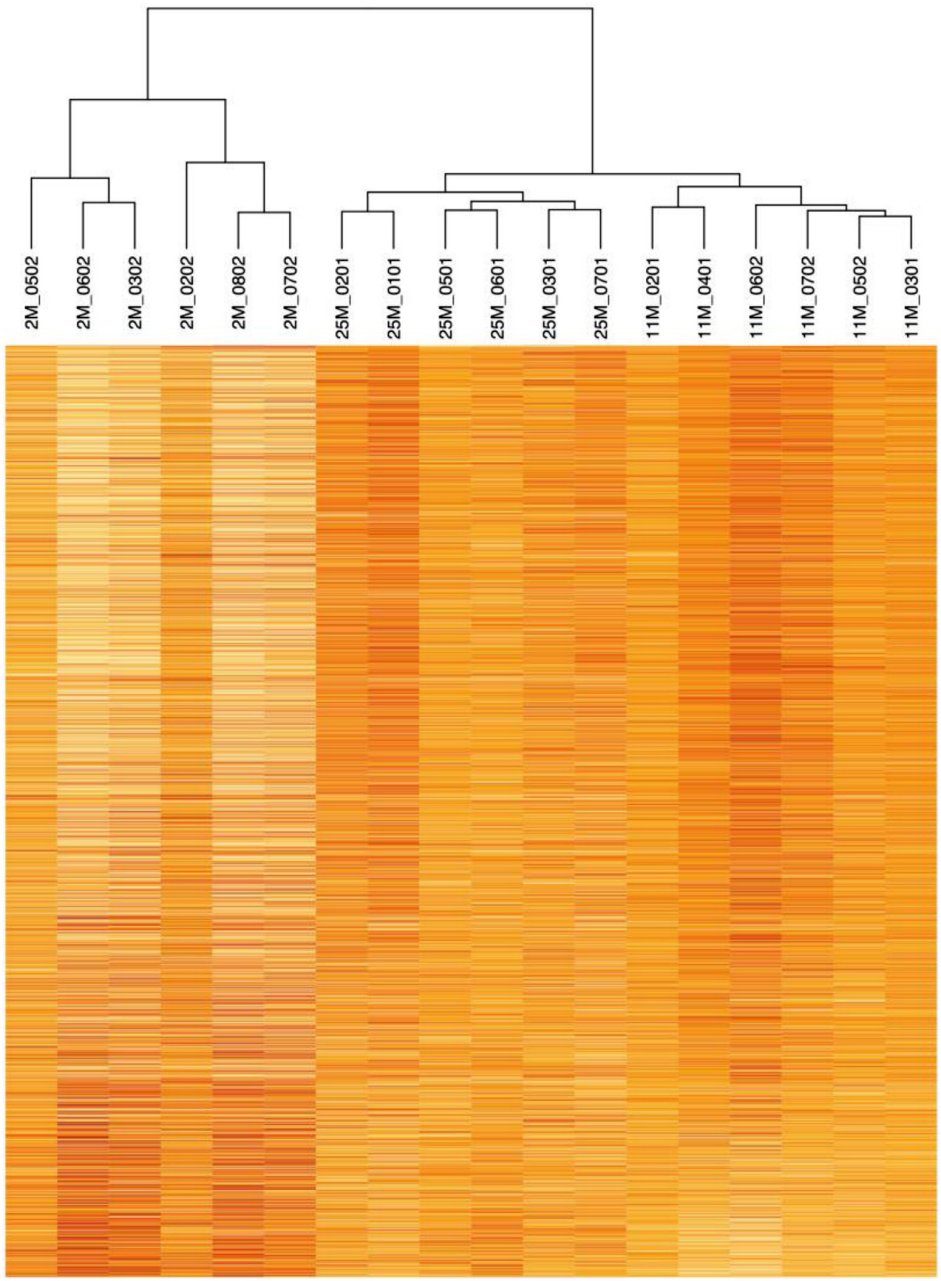
Heat-map illustrating the differentially expressed genes (P<0.05) between mice at different age groups. This heatmapwas build in base R, using the *heatmap() function* with no parameter. This function reorders both variables and observations using a clustering algorithm that computes the distance between each pair of rows and columns and try to order them by similarity. The dendrograms corresponding to mouse clustering is provided above the heatmap

The data includes eighteen .CEL files obtained after scanning mouse exon arrays available at the following address: https://www.ncbi.nlm.nih.gov/geo/query/acc.cgi?acc=GSE136266 and four .xls files presented as supplementary data (Table S1, Table S2, Table S3 and Table S4). Table S1 provides 15300 normalized mRNA levels for each mouse. Each file has two pages. The first page is labelled “Metadata template”; this page presents the subject area, how the data has been acquired, a short description of the model, the contributors, the samples. The second page contains the data. The first column of the data corresponds to an ordered number; the second column corresponds to a cluster identification number (column “Transcripts Cluster Id”); the third column corresponds to a brief description of each gene; the fourth and fifth columns cover the corresponding gene symbols (column “gene symbol and gene symbol1”). The other columns correspond to the normalized intensity data of each gene for each of the 18 mice. These data are illustrated by a heat-map (Fig. 1). Tables S2, S3 and S4 highlight the significantly different genes between young, mature adult and aged groups (Table S2 between stages 2 and 11 months old; Table S3 between stages 2 and 25 months old; Table S4 between stages 11 and 25 months old). The first column of each file corresponds to the transcript cluster id, the second one to the gene symbol, the third one to the gene description, the fourth to the significance (significance for p<0.05) and the fifth to the fold change (FC) that shows the amplitude of the difference between the stages.

## 2 Experimental Design, Materials and Methods

### 2.1 Animals and samples

**Fig. 2:**
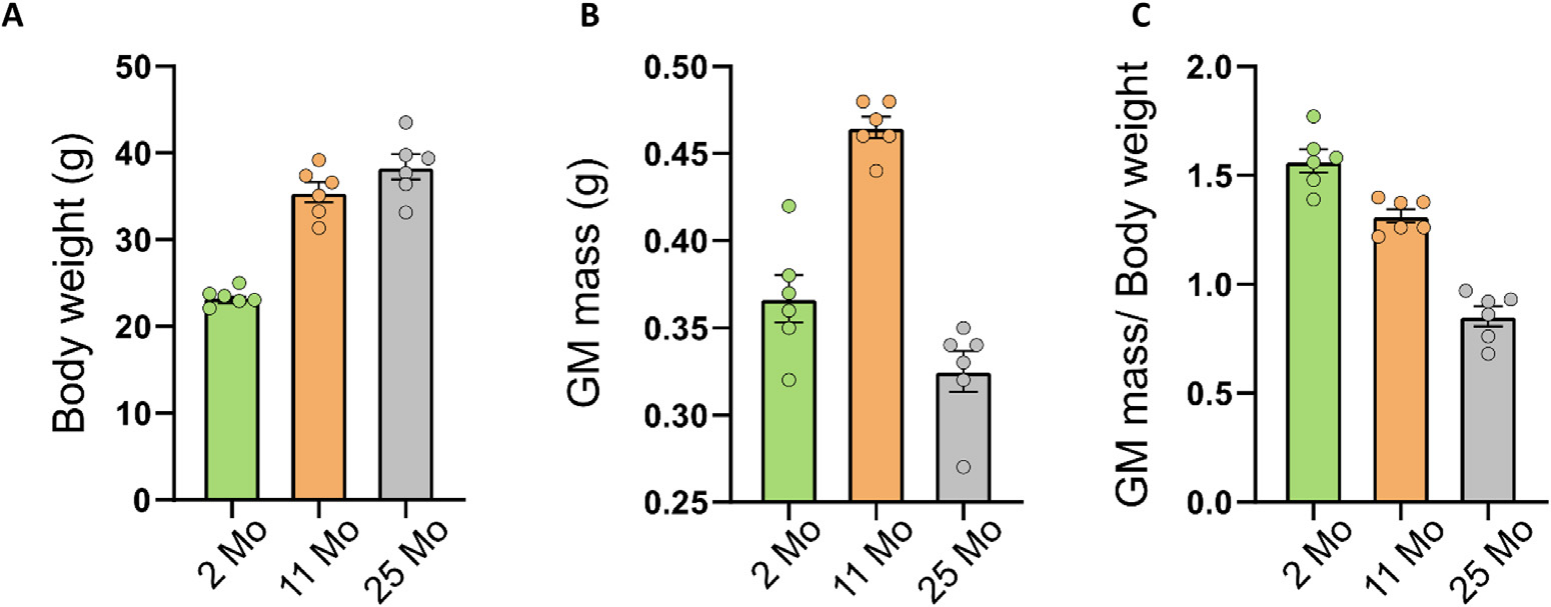
Histograms of (A) body weight (g), (B) *Gastrocnemius* (GM) muscle mass (g) and (C) GM muscle mass/Body weight from C57BL/6 mice at 2, 11 and 25 month (Mo) of age. All data points were shown on the histograms for each age.

Eighteen C57BL/6 mice of 2, 11 and 25 months old (6 per age group) were used in this study. The main objective being to highlight whether there were genes or biological processes specific of ageing (compared to young age or adulthood), we needed samples representative of these stages. To choose them we relied to our previous results [1] which showed that both body and *Gastrocnemius* (GM) muscle weights increased a lot between 1 and 8 months, then they stabilized between 8 and 16 months, after which the GM weight decreased while the body weight remained stable. We obtained the same trends with the animals of the present study, i.e. an increase of the body weight and GM muscle mass between 2 and 11 months, followed by a stabilization of body weight and a decrease of the GM muscle mass. This translated into a progressive decrease of the ratio GM muscle mass on body weight between 2 and 25 months (Fig. 2A, B, C). As we hypothesized that the changes of muscle weight were representative of young, mature or aged states and we were hoping to complete our previous data, we chose the stages included in the phases of significant muscle weight changes, i.e. 2, 11 and 25 months. In addition, we chose to work with only one gender (male mice) to avoid the variability related to this parameter. We chose the male mice because our experience with the mouse C57BL/6 strain had shown that the effects of age (for example, loss of activity, weight) were more marked in males at the end of life. This result has been confirmed by Tran *et al.* [2].

Animals were obtained from the Laboratory Animal Centre of the National University of Singapore and raised in the veterinarian-staffed Laboratory Animal Facility at Nanyang Technological University (NTU) following the procedure of the Institutional Animal Care-and-Use Committee.

Mice were housed in a temperature (22 ± 1 °C)- and humidity (50–70%)-controlled facility, with a 12:12 h light–dark cycle, and food (SAFE, Singapore, Singapore) and water were provided *ad libitum*. Mice were either 2, 11 or 25 months of age, corresponding to young adult, mature adult or advanced old mice, respectively. Mice were killed by cervical dislocation after CO_2_ anaesthesia and weighted. *Gastrocnemius* muscle (GM) were rapidly removed (within 10 min.) from both hind limbs, weighted and snap-frozen in liquid nitrogen for subsequent extraction of total RNA. Body weight, GM muscle mass and ratio of GM muscle mass on Body weight were illustrated in Fig. 2A, B, C.

### 2.2 RNA extraction

For each mouse, RNA from *Gastrocnemius* muscle (GM) of both legs was isolated using a Tissue Ruptor and RNeasy Fibrous Tissue Mini Kit (Qiagen). Integrity of RNA samples was verified using an Agilent Bioanalyser with the RNA 6000 Nano labchip® kit). Concentration and purity was determined with a Nanodrop ND-1000 spectrophotometer. Samples with a 28S/18S ratio <1.0 were excluded. On average, the RIN value of 6.5 was obtained, which is normal for RNA samples from animal tissues [3].

### 2.3 Exon array data collection

**Fig. 3:**
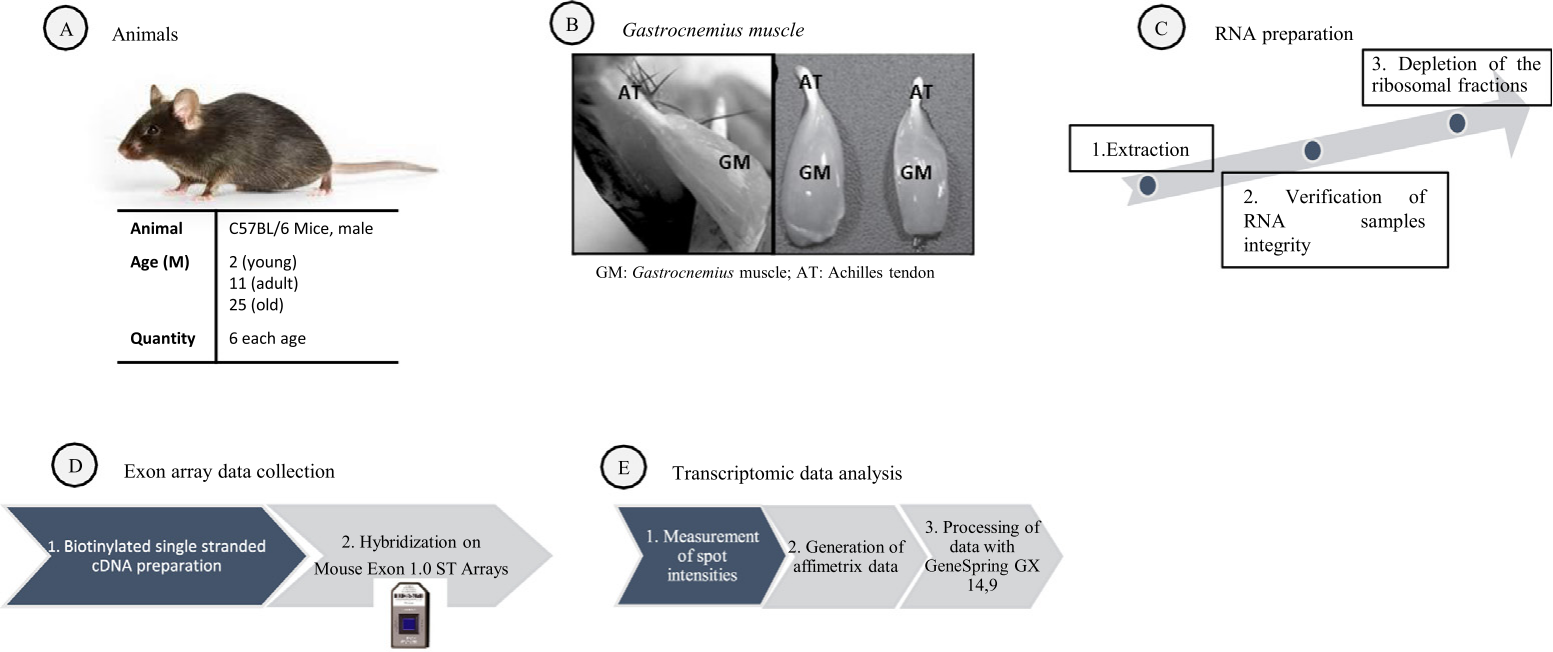
(A) Animal used in this study. (B) For each animal, *Gastrocnemius* muscle (GM) from both left and right legs were dissected. (C) Total RNA was extracted from GM muscle (D) Hybridization were performed with Mouse Exon 1.0 ST arrays. (E) Transcriptomic data were analysed.

The depletion of the ribosomal fractions was processed using 2 µg of total RNA and magnetic beads from Ribominus Kit (Invitrogen). Biotinylated single stranded cDNA were prepared from 400 ng of depleted total RNA according to the Affymetrix WT protocol (Ambion WT Expression kit). Following fragmentation and terminal labeling, 5.5 µg of single stranded cDNA were hybridized for 16 h at 45 °C and 60 rpm on GeneChip® Mouse Exon 1.0 ST Array in the Affymetrix Oven 645. GeneChips were washed and stained in the Affymetrix Fluidics Station 450 with Hybridization Wash and Stain Affymetrix kit. GeneChips were scanned using the Affymetrix GeneChip Scanner 3000 7G, and data were generated with Affymetrix Expression Console v 1.2.1 software using robust multiarray average (RMA) algorithm. Parameter setting and summarization was performed using RMA algorithm. “Core” list was sourced from metaprobeset list (available on Expression Console by Affymetrix) and comprised 17,958 transcript clusters from RefSeq and full length GenBank mRNAs. Affymetrix data included 18 .CEL intensity files. All steps of experimental protocol were summarized in Fig. 3A, B, C, D, E.

### 2.4 Transcriptomic data analysis

**Fig. 4:**
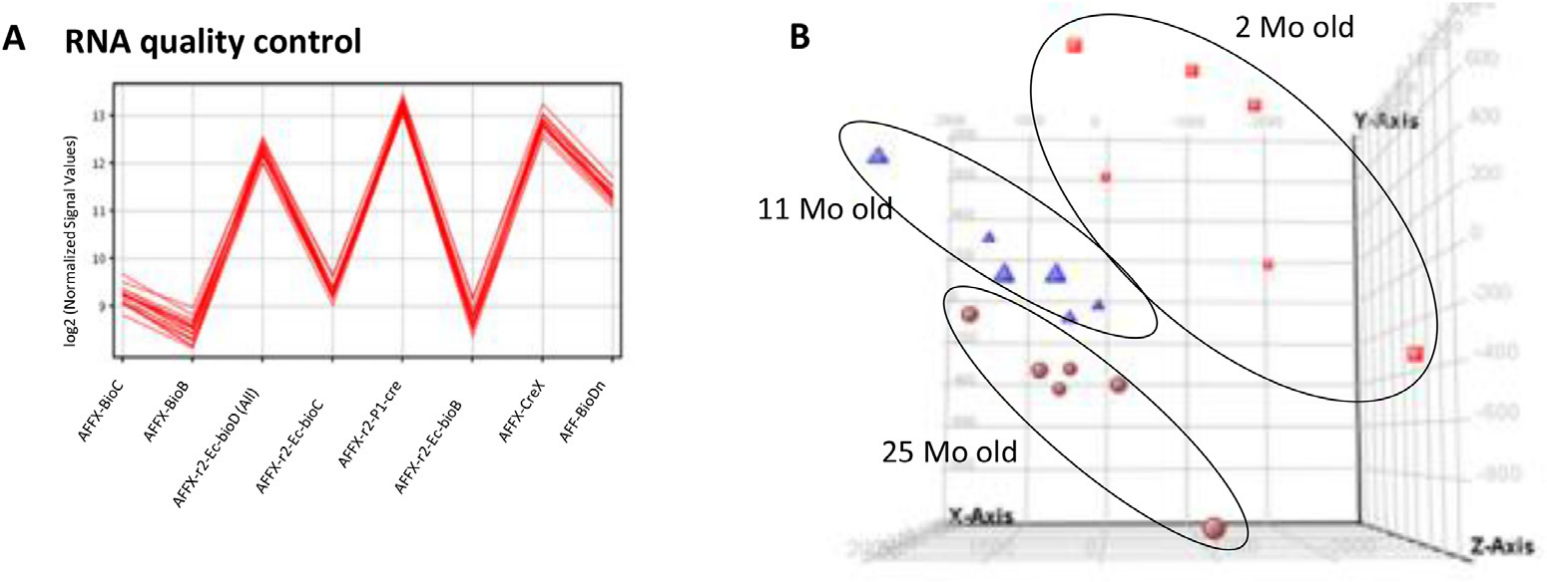
A. Quality controls of hybridizations were realized using the Affymetrix probes AFFX-BioC, AFFX-BioB, AFFX-r2-Ec-bioD, AFFX-r2-Ec-bioC, AFFX-r2-P1-cre, AFFX-r2-Ec-bioB, AFFX-CreX and AFF-BioDn. B. Eighteen arrays data were projected in 3D spaces comprising PC1, PC2 and PC3 as x-, y- and z-axis. PCA analysis, red cubes, blue triangles and brown balls were used to represent array data from mice at 2, 11 and 25M, respectively.

Affymetrix data files were processed using GeneSpring GX 14.9 (Agilent Technologies). We have added parameter “age” to help define the grouping and replicate structure of the experiment. Quality control was performed to examine the data and if no ambiguous samples had to be removed. Quality control started from hybridization control using 8 internal probes to depict hybridization quality (AFFX-BioC, AFFX-BioB, AFFX-r2-Ec-bioD, AFFX-r2-Ec-bioC, AFFX-r2-P1-cre, AFFX-r2-Ec-bioB, AFFX-CreX and AFF-BioDn), which indicated similar hybridization signals between samples (Fig. 4A). Next, 3D-PCA was performed to displays the correlation of expression. PC1, PC2 and PC3 explained 54%, 21% and 13% of total variance, respectively (Fig. 4B). Differentially expressed entities were then reported after performing one-way ANOVA followed by pairwise comparisons using Tukey HSD post-hoc test. P-values were calculated asymptotically and Benjamini-Hochberg (B-H) false discovery rate (FDR) was selected to correct the p-values. The corrected p-value <0.05 was used to determine statistically significant differential expression of genes between mice at different age groups (Table S2-S4). This method has been previously published by Gueugneau *et al.* (2021) [4].

## Ethical Statements

All animal procedures were conducted in compliance with the NTU Institutional Animal Care-and-Use Committee (IACUC) guidelines (NTU-IACUC Ref: IACUC ARF SBS/NIE-A 00 36). No animal experiments were performed. Only tissues from the male C57BL/6 mice were used in this study*.*

## CRediT authorship contribution statement

**Anne Listrat:** Conceptualization, Data curation, Formal analysis, Investigation, Methodology, Project administration, Resources, Software, Supervision, Validation, Visualization, Writing – original draft, Writing – review & editing. **Kheng Lim Goh:** Conceptualization, Funding acquisition, Software, Supervision, Writing – review & editing. **Céline Jousse:** Conceptualization, Supervision, Writing – review & editing. **Jérémy Tounayre:** Conceptualization, Writing – review & editing. **Huijuan Wang:** Conceptualization, Data curation, Formal analysis, Investigation, Methodology, Project administration, Software, Validation, Visualization, Writing – review & editing. **Kijoon Lee:** Conceptualization, Funding acquisition, Investigation, Methodology, Supervision, Writing – review & editing. **Daniel Béchet:** Conceptualization, Investigation, Methodology, Resources, Software, Supervision, Writing – review & editing.

## Declaration of Competing interest

The authors declare that they have no known competing financial interests or personal relationships that could have appeared to influence the work reported in this paper.

## Data Availability

Dataset on transcriptome signature of skeletal muscle of young, adult and aged mice (GSE136266) (Original data) (NCBI GEO (Gene Expression Omnibus)).

## Appendix Supplementary materials

Image, application 1

Image, application 2

Image, application 3

Image, application 4
